# Anti-Inflammatory and Barrier-Related Effects of *Bidens bipinnata* L. Fruit Ethanol Extract in an MC903-Induced AD-like Dermatitis Mouse Model and LPS-Stimulated RAW 264.7 Cells

**DOI:** 10.3390/ijms27135717

**Published:** 2026-06-24

**Authors:** Jinhu Peng, Yanfeng Ren, Jimi Lee, Soyeon Kim, Jung-Hoon Kim, Hyungwoo Kim

**Affiliations:** Division of Pharmacology, School of Korean Medicine, Pusan National University, Yangsan 50612, Gyeongnam, Republic of Korea; pengjinhu40@gmail.com (J.P.); zdhf15@163.com (Y.R.);

**Keywords:** *Bidens bipinnata*, atopic dermatitis, barrier-related proteins, inflammation

## Abstract

Atopic dermatitis (AD) is a chronic inflammatory dermatosis driven by skin barrier impairment and immune dysregulation. This study aimed to investigate the anti-inflammatory and barrier-related effects of the ethanol extract of *Bidens bipinnata* L. fruits (EEBB) in a calcipotriol (MC903)-induced AD-like dermatitis mouse model and lipopolysaccharide (LPS)-stimulated RAW 264.7 macrophages. In vivo, repeated topical application of EEBB (60, 180, and 600 μg/day) significantly attenuated MC903-induced AD-like clinical symptoms, skin weight, and erythema index. Notably, EEBB significantly improved skin hydration-related parameters, including relative skin hydration readings and the post-application moisture retention profile, and partially restored filaggrin and loricrin expression in lesional skin, whereas dexamethasone showed limited effects on these hydration-related parameters under the present conditions. Histopathologically, EEBB ameliorated epidermal lesions and reduced inflammatory cell infiltration. Mechanistically, EEBB suppressed the levels of pro-inflammatory (TNF-α, IFN-γ) and Th2 (IL-4, IL-5) cytokines in lesional skin. In vitro, EEBB significantly inhibited the production of nitric oxide (NO) and prostaglandin E_2_ (PGE_2_), and downregulated inducible nitric oxide synthase (iNOS) and cyclooxygenase-2 (COX-2) protein expression in RAW 264.7 cells. These effects were associated with inhibited phosphorylation of JNK and p38 MAPK, with no marked effect on ERK phosphorylation under the present conditions. In conclusion, EEBB effectively alleviated AD-like dermatitis, accompanied by improved skin hydration and restoration of barrier-related protein expression, attenuation of local inflammatory responses, and targeted inhibition of the MAPK signaling pathway.

## 1. Introduction

Atopic dermatitis (AD) is a common chronic relapsing inflammatory skin disease characterized by pruritus, epidermal barrier dysfunction, and immune dysregulation [1]. Disruption of the epidermal barrier facilitates the penetration of environmental allergens, irritants, and microbes into the skin, thereby amplifying cutaneous inflammation and contributing to disease chronicity [2,3,4]. Because barrier impairment and inflammatory responses interact in a mutually reinforcing manner, both anti-inflammatory activity and barrier-supportive effects are important therapeutic considerations in AD [5]. Although topical corticosteroids remain a mainstay of treatment, their prolonged or repeated use may be limited by adverse effects such as skin atrophy and other barrier-unfavorable changes [6]. Therefore, there is increasing interest in safer therapeutic candidates, particularly natural products that can simultaneously attenuate inflammation and support barrier-related skin homeostasis.

*Bidens bipinnata* L., commonly known as Spanish needles, is an annual herbaceous plant of the Asteraceae family that is widely distributed across tropical, subtropical, and temperate regions [7,8]. In East Asian traditional medicine, including Korea, China, and Japan, the whole plant has long been used for inflammatory and infectious conditions and has also been applied topically to traumatic injury, snake and insect bites [9,10,11]. According to traditional medical theory, it is characterized by a cooling nature and is classified as a pharmacologically active remedy to clear away superficial heat and toxic materials, eliminate wind-dampness, and resolve blood stasis [11,12].

The pharmacological potential of *B. bipinnata* has been attributed to its diverse bioactive constituents, including flavonoids such as quercetin, okanin, and butein, as well as polyacetylenes and phenolic acids [7,8,13,14]. Previous in vitro studies have shown that compounds derived from Bidens species exert anti-inflammatory effects by modulating macrophage activation, including inhibition of nitric oxide (NO) production, downregulation of inducible nitric oxide synthase (iNOS), and suppression of pro-inflammatory cytokines, including tumor necrosis factor (TNF)-α, interleukin (IL)-1β, and IL-6, through regulation of the nuclear factor kappa B (NF-κB) and mitogen-activated protein kinase (MAPK) signaling pathways [15,16,17].

Despite these reported activities, the role of *B. bipinnata* fruits in AD-related skin inflammation and barrier-associated alterations remains insufficiently explored. In particular, the fruit part has been relatively underexplored, and its protective effects on AD-relevant inflammatory and barrier-associated skin changes remain unclear. AD pathogenesis involves inflammatory mediator production, cytokine dysregulation, and epidermal barrier impairment, and these features are mechanistically relevant to the reported pharmacological activities of *B. bipinnata*. Therefore, the known anti-inflammatory properties of this plant provide a rationale for investigating its fruit extract in the context of AD-like dermatitis.

Accordingly, the present study investigated whether the ethanol extract of *B. bipinnata* fruits (EEBB) could alleviate AD-like dermatitis through anti-inflammatory effects and improvement in hydration- and barrier-associated skin changes. To address this question, we evaluated the effects of *B. bipinnata* fruits in an MC903-induced AD-like dermatitis mouse model and in lipopolysaccharide (LPS)-stimulated RAW 264.7 macrophages. Specifically, we examined its effects on clinical and histopathological skin changes, hydration- and barrier-related parameters, inflammatory cytokines in lesional skin, and inflammatory mediator-associated signaling pathways in vitro.

## 2. Results

### 2.1. EEBB Alleviated AD-like Symptoms and Reduced Clinical Skin Lesion Scores

**Figure 1:**
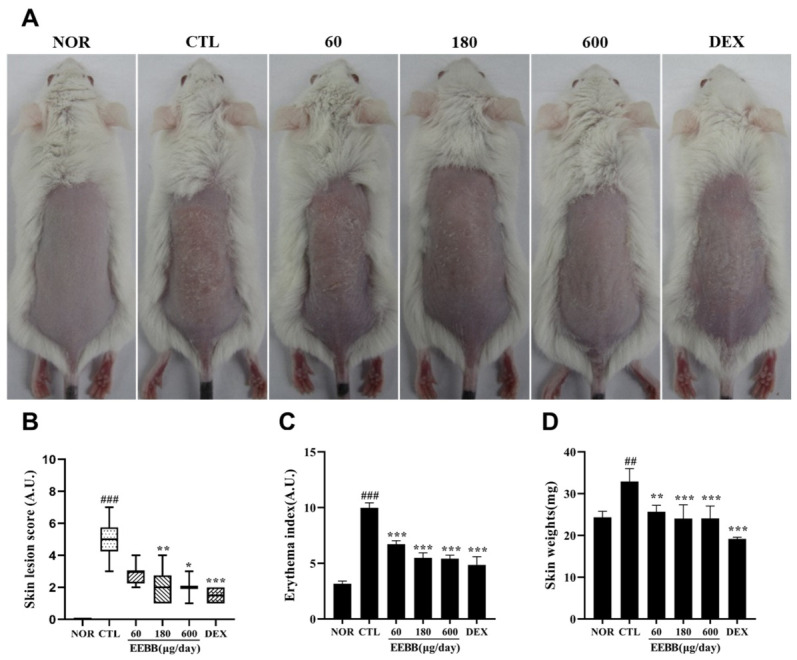
Effects of EEBB on skin lesions, erythema index, and skin weight in mice with AD-like dermatitis. (**A**) Representative photographs of dorsal skin lesions on Day 16. (**B**) Skin lesion severity in each group was evaluated using a 4-point severity scoring system. (**C**) Quantitative analysis of erythema index was performed using a skin colorimeter. (**D**) Skin weight was measured on Day 16. NOR, normal group; CTL, MC903-induced AD-like dermatitis group; 60, 60 μg/day EEBB; 180, 180 μg/day EEBB; 600, 600 μg/day EEBB; DEX, 150 μg/day dexamethasone. A.U., arbitrary units; EEBB, ethanol extract of *B. bipinnata*. Values are expressed as median (IQR) for skin lesion scores and mean ± SD for erythema index and skin weight. ^##^ *p* < 0.01 and ^###^ *p* < 0.001 vs. NOR; * *p* < 0.05, ** *p* < 0.01 and *** *p* < 0.001 vs. CTL.

Repeated topical application of MC903 to the dorsal skin induced AD-like skin lesions, manifested by prominent symptoms including scaling, erythema, roughness, and fissuring. Treatment with EEBB effectively attenuated these skin lesions (Figure 1A). Compared with the AD-like dermatitis model group (CTL), clinical skin lesion scores were significantly lower in the 180 and 600 μg/day EEBB-treated groups (Figure 1B). In addition, the erythema index was significantly reduced in all EEBB-treated groups and in the DEX group compared with the CTL group (*p* < 0.001) (Figure 1C). Furthermore, skin weight was significantly reduced in all three EEBB-treated groups and in the DEX-positive control group compared with the CTL group (*p* < 0.01) (Figure 1D).

### 2.2. EEBB Improved Skin Hydration-Related Parameters in Mice with AD-like Dermatitis

**Figure 2:**
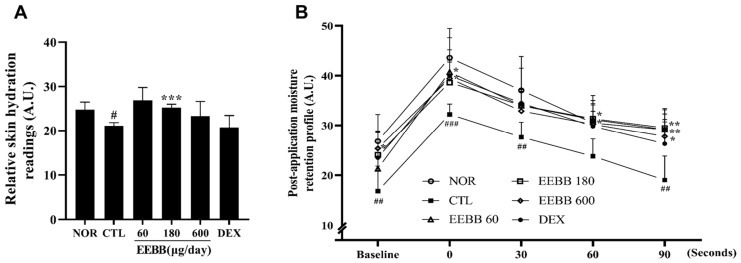
Effects of EEBB on skin hydration-related measurements in mice with AD-like dermatitis. (**A**) Relative skin hydration readings; (**B**) Post-application moisture retention profile. NOR, normal group; CTL, MC903-induced AD-like dermatitis group; 60, 60 μg/day EEBB; 180, 180 μg/day EEBB; 600, 600 μg/day EEBB; DEX, 150 μg/day dexamethasone. A.U., arbitrary units; EEBB, ethanol extract of *B. bipinnata*. Values are expressed as mean ± SD. ^#^ *p* < 0.05, ^##^ *p* < 0.01 and ^###^ *p* < 0.001 vs. NOR; * *p* < 0.05, ** *p* < 0.01 and *** *p* < 0.001 vs. CTL.

Compared with the NOR group, the CTL group exhibited significantly reduced relative skin hydration readings and a diminished post-application moisture retention profile. However, treatment with 180 μg/day EEBB significantly increased skin hydration compared with the CTL group (*p* < 0.001) (Figure 2A). Additionally, all three EEBB-treated groups showed varying degrees of improvement in the post-application moisture retention profile, with the 180 μg/day group demonstrating the most pronounced effect among the three doses (*p* < 0.001) (Figure 2B). Notably, DEX treatment had no significant effect on either of these hydration-related parameters (*p* > 0.05).

### 2.3. EEBB Ameliorated Histopathological Changes and Reduced Inflammatory Cell Infiltration in Lesional Skin

**Figure 3:**
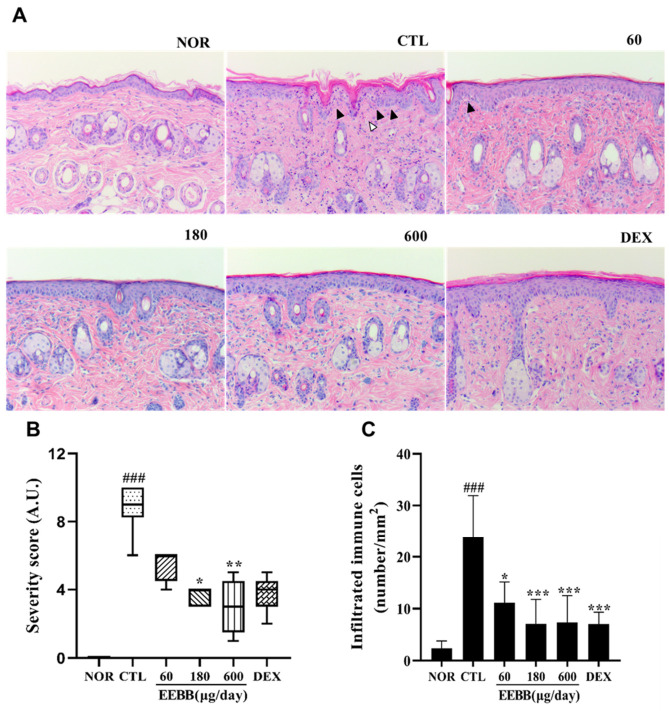
Effects of EEBB on histopathological changes in mice with AD-like dermatitis. (**A**) Representative H&E-stained skin sections of epidermal and dermal morphology (original magnification, ×100). Black arrowheads indicate disruption of the basal layer. White arrowheads indicate newly formed blood vessels. (**B**) Histopathological severity scores. (**C**) Immune cell counts. NOR, normal group; CTL, MC903-induced AD-like dermatitis group; 60, 60 μg/day EEBB; 180, 180 μg/day EEBB; 600, 600 μg/day EEBB; DEX, 150 μg/day dexamethasone. EEBB, ethanol extract of *B. bipinnata*. Values are expressed as median (IQR) for histopathological severity scores and mean ± SD for immune cell counts. ^###^ *p* < 0.001 vs. NOR, * *p* < 0.05, ** *p* < 0.01 and *** *p* < 0.001 vs. CTL.

MC903 successfully induced typical AD-like lesions characterized by epidermal structural alterations and dermal inflammatory cell infiltration, predominantly lymphocytic. Specific manifestations included moderate epidermal hyperkeratosis, mild-to-moderate acanthosis with scattered lymphocytic infiltration, and focal spongiosis (characterized by intercellular edema and widened intercellular spaces). Additionally, we observed blurring of the dermal-epidermal junction and disruption of the basal layer (indicated by black arrowheads), along with a diffuse distribution of inflammatory cells extending from the papillary dermis to the superficial reticular dermis and perivascular regions. Furthermore, a small number of eosinophils (identified by their eosinophilic cytoplasm and bilobed nuclei) as well as angiogenesis (indicated by white arrowheads) were evident (Figure 3A).

In contrast, treatment with all three doses of EEBB, as well as DEX, ameliorated epidermal structural abnormalities and appeared to reduce inflammatory cell infiltration and angiogenic changes (Figure 3A). Quantitatively, compared with the NOR group, the histological severity scores and immune cell counts in the CTL group were significantly elevated (*p* < 0.001). However, the histological severity score was significantly reduced in the 180 and 600 μg/day EEBB-treated groups compared with the CTL group, whereas the DEX group did not show a significant difference (Figure 3B). In addition, infiltrated immune cell counts were significantly reduced in all treatment groups compared with the CTL group, with *p* < 0.05 for the 60 μg/day EEBB-treated group and *p* < 0.001 for the 180 and 600 μg/day EEBB-treated groups and the DEX group (Figure 3C).

### 2.4. EEBB Restored Epidermal Barrier-Related Protein Expression in Lesional Skin

**Figure 4:**
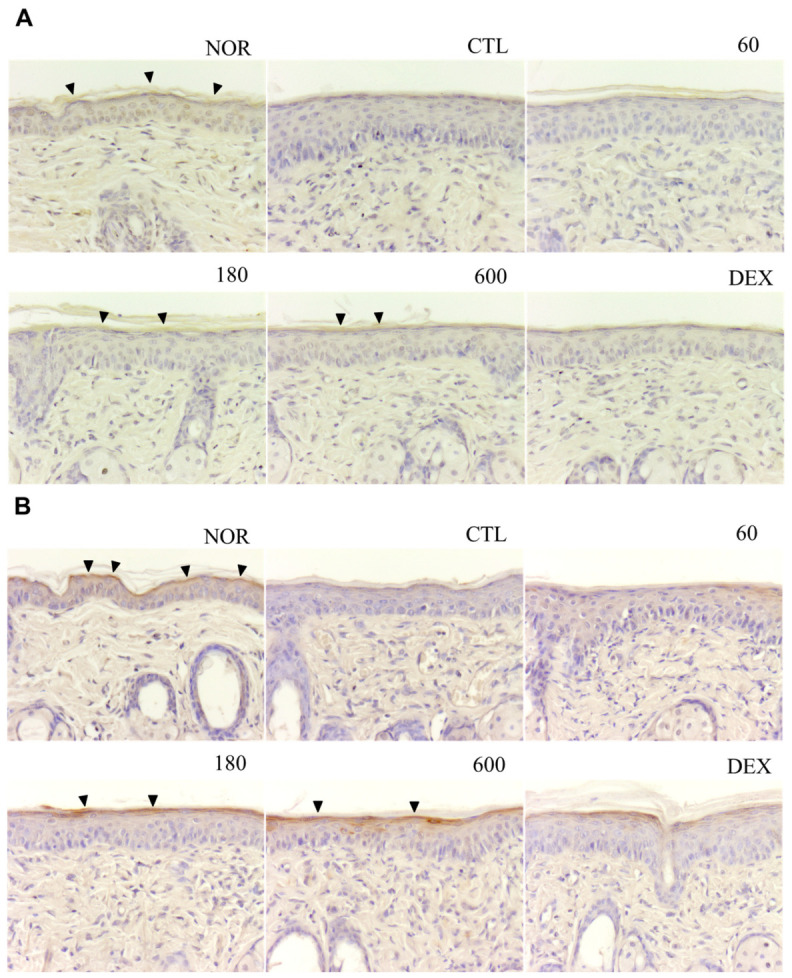
Effects of EEBB on filaggrin and loricrin expression in lesional skin. (**A**) Filaggrin and (**B**) loricrin (original magnification, ×200). Black arrowheads indicate the brown-stained regions showing filaggrin or loricrin immunoreactivity. NOR, normal group; CTL, MC903-induced AD-like dermatitis group; 60, 60 μg/day EEBB; 180, 180 μg/day EEBB; 600, 600 μg/day EEBB; DEX, 150 μg/day dexamethasone.

Immunohistochemical analysis revealed that the expression of filaggrin and loricrin was markedly reduced in the dorsal skin of MC903-treated mice compared with that in the NOR group. Topical treatment with EEBB partially restored the expression pattern of filaggrin and loricrin in lesional skin, suggesting a protective effect on barrier-associated epidermal structures. In contrast, the effect of dexamethasone on these markers was limited (Figure 4).

### 2.5. EEBB Reduced the Levels of Pro-Inflammatory and Th2 Cytokines in Lesional Tissue

**Figure 5:**
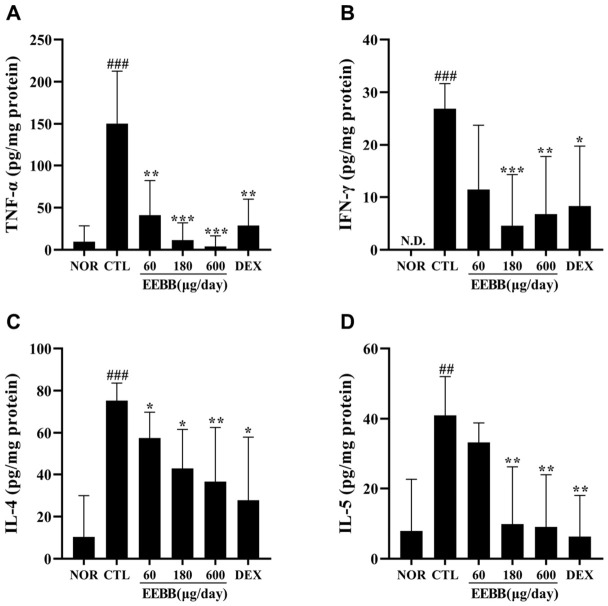
Effects of EEBB on the levels of TNF-α, IFN-γ, IL-5, and IL-4 in dorsal skin tissue. (**A**) TNF-α; (**B**) IFN-γ; (**C**) IL-4; (**D**) IL-5. NOR, normal group; CTL, MC903-induced AD-like dermatitis group; 60, 60 μg/day EEBB; 180, 180 μg/day EEBB; 600, 600 μg/day EEBB; DEX, 150 μg/day dexamethasone. EEBB, ethanol extract of *B. bipinnata*; N.D., not detected. Values are expressed as mean ± SD. ^##^ *p* < 0.01 and ^###^ *p* < 0.001 vs. NOR; * *p* < 0.05, ** *p* < 0.01 and *** *p* < 0.001 vs. CTL.

Repeated topical application of MC903 significantly increased the levels of the pro-inflammatory cytokines TNF-α and IFN-γ, and the Th2 cytokines IL-4 and IL-5, compared with the NOR group (*p* < 0.001). Topical treatment with EEBB at a dose of 60 μg/day significantly reduced the levels of TNF-α and IL-4 compared with the CTL group (*p* < 0.05). At higher doses (180 and 600 μg/day), EEBB significantly suppressed the levels of all four cytokines. Similarly, DEX treatment significantly reduced the levels of all four cytokines (Figure 5).

### 2.6. EEBB Reduced the Levels of Inflammatory Mediators NO and PGE_2_ in LPS-Induced RAW 264.7 Cells

**Figure 6:**
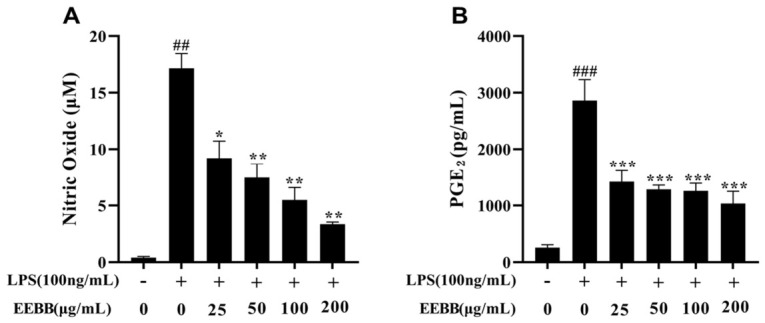
Effects of EEBB on NO and PGE_2_ Levels in RAW 264.7 cells. Cells were stimulated with LPS (100 ng/mL). (**A**) NO, nitric oxide; (**B**) PGE_2_, prostaglandin E_2_. EEBB, ethanol extract of *B. bipinnata.* Values are expressed as mean ± SD. ^##^ *p* < 0.01 and ^###^ *p* < 0.001 vs. unstimulated control; * *p* < 0.05, ** *p* < 0.01 and *** *p* < 0.001 vs. LPS control (n = 3).

Stimulation with LPS (100 ng/mL) elicited a marked inflammatory response, as evidenced by significantly elevated levels of NO (*p* < 0.01) (Figure 6A) and prostaglandin E_2_ (PGE_2_) (*p* < 0.001) (Figure 6B) relative to the unstimulated control group. However, pretreatment with EEBB exerted a dose-dependent inhibitory effect on both mediators. Specifically, EEBB significantly suppressed NO accumulation (*p* < 0.05), with the most pronounced inhibition observed at the highest concentration of 200 μg/mL. Likewise, EEBB at all tested concentrations (25, 50, 100, and 200 μg/mL) significantly reduced LPS-induced PGE_2_ production (*p* < 0.001).

### 2.7. EEBB Inhibited LPS-Induced iNOS and COX-2 Expression and Suppressed p38 and JNK Phosphorylation in RAW 264.7 Cells

**Figure 7:**
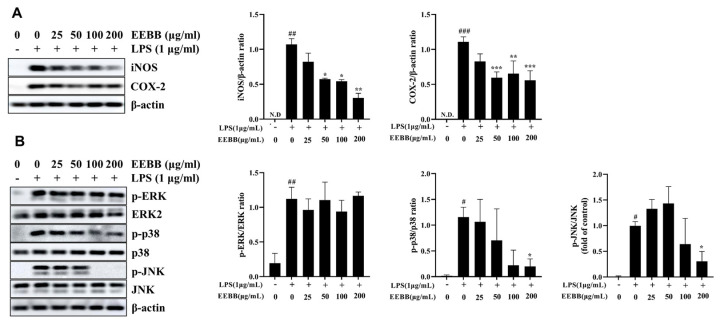
Effects of EEBB on iNOS and COX-2 protein expression and MAPK signaling pathways in RAW 264.7 Cells. (**A**) iNOS and COX-2 protein expression. RAW 264.7 cells were pretreated with EEBB for 8 h, followed by LPS (1 μg/mL) stimulation for 20 h. (**B**) MAPK signaling pathways. Cells were pretreated with EEBB for 10 h and then stimulated with LPS (1 μg/mL) for 30 min. Total protein was extracted and analyzed by Western blot. β-actin was used as an internal loading control. iNOS, inducible nitric oxide synthase; COX-2, cyclooxygenase-2; MAPK, mitogen-activated protein kinase. Values are expressed as mean ± SD. ^#^ *p* < 0.05, ^##^ *p* < 0.01 and ^###^ *p* < 0.001 vs. unstimulated control; * *p* < 0.05, ** *p* < 0.01 and *** *p* < 0.001 vs. LPS control (n = 3).

To further investigate the molecular basis of these anti-inflammatory effects, we performed Western blot analysis in RAW 264.7 cells stimulated with 1 μg/mL LPS to assess the expression levels of iNOS and cyclooxygenase-2 (COX-2). As shown in Figure 7A, LPS stimulation markedly upregulated both iNOS and COX-2 protein levels relative to the unstimulated control group. Pretreatment with EEBB attenuated the LPS-induced elevation of both proteins, as confirmed by densitometric analysis. Furthermore, EEBB significantly inhibited the LPS-induced phosphorylation of p38 (p38 mitogen-activated protein kinase) and JNK (c-Jun N-terminal kinase) at the highest tested concentration (200 μg/mL), whereas it did not significantly affect ERK (extracellular signal-regulated kinase) phosphorylation (Figure 7B).

## 3. Discussion

In the present study, topical EEBB alleviated MC903-induced AD-like dermatitis in mice, as evidenced by reduced clinical lesion severity, erythema index, skin weight, histopathological abnormalities, and inflammatory cytokine levels in lesional skin. EEBB was also associated with increased relative skin hydration readings, a more favorable post-application moisture retention profile, and restored expression of the epidermal differentiation-related proteins filaggrin and loricrin. In parallel, EEBB suppressed NO and PGE_2_ production, downregulated iNOS and COX-2 expression, and inhibited JNK and p38 phosphorylation in LPS-stimulated RAW 264.7 cells. Collectively, these findings suggest that EEBB exerts anti-inflammatory effects while supporting recovery of barrier-related epidermal changes in this AD-like dermatitis model.

Because epidermal barrier impairment is a central feature of AD pathophysiology, the barrier-associated findings of the present study are of particular interest. In MC903-treated mice, skin hydration and the post-application moisture retention profile were reduced, together with decreased expression of filaggrin and loricrin in lesional skin. EEBB treatment partially reversed these alterations and was accompanied by improvement in macroscopic disease severity, including reduced scaling and erythema. Given the important roles of filaggrin and loricrin in epidermal differentiation and stratum corneum integrity [5], the restored expression of these proteins suggests that EEBB may help normalize barrier-associated epidermal alterations in this model. Nevertheless, because direct functional assessments of barrier integrity, such as transepidermal water loss (TEWL) or permeability assays, were not performed, these findings should be interpreted as evidence of hydration-related improvement and restoration of barrier-associated protein expression, rather than as definitive evidence of functional barrier recovery.

Histopathological examination further supported these observations, showing that EEBB mitigated MC903-induced epidermal hyperkeratosis, spongiosis, and inflammatory cell infiltration. These tissue-level improvements are consistent with the reductions in clinical lesion severity and erythema, suggesting that EEBB attenuated both macroscopic and microscopic manifestations of AD-like skin inflammation. In the present study, skin weight was included as a supplementary gross parameter of the biopsy specimen. We acknowledge that this measure is not specific to epidermal change and may be influenced by multiple factors, including overall tissue swelling, edema, and dermal inflammatory cell infiltration. Therefore, the skin weight findings should be interpreted cautiously and regarded as supportive rather than as a direct histological indicator.

Another noteworthy finding was the difference between EEBB and dexamethasone in hydration-related outcomes. Although DEX effectively reduced inflammatory parameters, it did not significantly improve relative skin hydration readings or the post-application moisture retention profile under the present conditions, consistent with the findings reported by Son et al. [18]. By contrast, EEBB showed anti-inflammatory activity together with improvement in hydration-related measurements and partial restoration of filaggrin and loricrin expression. These results suggest that EEBB may exert broader skin-protective effects than anti-inflammatory suppression alone, particularly with respect to hydration-related and barrier-associated epidermal changes.

Interestingly, although EEBB improved AD-related outcomes overall, the intermediate dose (180 μg/day) appeared to show greater efficacy than the high dose (600 μg/day) in several parameters, particularly those related to relative skin hydration readings, the post-application moisture retention profile, and some histological readouts. However, this tendency was not consistently observed across all measured endpoints, as some inflammatory parameters showed comparable responses between the 180 and 600 μg/day groups. Therefore, the present data suggest that the dose–response relationship of EEBB may not be strictly linear. Given that EEBB is a crude botanical extract containing multiple phytochemical constituents, its overall biological activity may reflect the combined and potentially dose-sensitive interaction of these components rather than a simple increase in efficacy with increasing dose. In this context, the intermediate dose may represent a more favorable range for certain outcomes. Because the present study was not designed to clarify the basis of this pattern, further studies will be needed to identify the active constituents, clarify component–effect relationships, and determine the optimal therapeutic window of EEBB.

Beyond barrier restoration, modulation of the immune microenvironment is also an important therapeutic consideration in AD. Immunologically, AD exhibits a biphasic inflammatory profile [19]. In both human AD and experimental AD-like dermatitis, Th2- and Th1-associated cytokine responses may coexist depending on disease phase and model context [20]. In the present study, EEBB effectively suppressed the levels of IL-4 and IL-5 in the lesional skin, indicating its capacity to attenuate Th2-associated inflammatory responses in this model. Because IL-4 is known to downregulate epidermal differentiation proteins such as filaggrin [4], the EEBB-mediated suppression of IL-4 may be mechanistically linked to the restored expression of filaggrin and loricrin observed in lesional skin (Figure 4). Simultaneously, EEBB significantly reduced the levels of the pro-inflammatory Th1 cytokines, TNF-α and IFN-γ. TNF-α contributes to epidermal dysfunction by attenuating the expression of barrier-related proteins, while IFN-γ synergistically acts on keratinocytes to aggravate cutaneous inflammation [21,22,23,24]. Interestingly, EEBB reduced both Th2-associated cytokines (IL-4 and IL-5) and Th1-associated cytokines (TNF-α and IFN-γ) measured in lesional skin, suggesting effects on multiple inflammatory axes relevant to this model. Taken together, these findings suggest that EEBB may improve both inflammatory and epidermal differentiation-related abnormalities in MC903-induced AD-like dermatitis.

Furthermore, our H&E staining results demonstrated that EEBB significantly attenuated the infiltration of immune cells, including lymphocytes and eosinophils, in the dermis. This effect may be associated with reduced cytokine-driven inflammatory cell recruitment, although the present study did not directly assess chemokines or trafficking-related mediators. Accordingly, this histological improvement is better interpreted as evidence of local anti-inflammatory activity rather than of a specific effect on cell-recruitment mechanisms.

In experimental AD-like dermatitis models, severe cutaneous inflammation is frequently accompanied by systemic immune alterations, prominently including splenomegaly [18,25]. In the present study, mice in the CTL group exhibited a marked increase in the spleen-to-body weight ratio compared with the NOR group, which may reflect systemic immune perturbation associated with severe cutaneous inflammation. Interestingly, while EEBB did not induce a significant reduction in the spleen-to-body weight ratio relative to the CTL group, DEX caused a profound and significant decrease (Supplementary data S1, Figure S1). This marked reduction in the DEX group is consistent with the known systemic immunosuppressive effects of corticosteroids [26]. Under the present experimental conditions, the lack of a comparable decrease in the EEBB-treated groups may suggest a systemic response pattern distinct from that of DEX; however, this interpretation should be made cautiously because systemic inflammatory or immune markers, such as serum TNF-α and IL-4, were not assessed. Therefore, the spleen index finding should be regarded as indirect and supplementary rather than definitive evidence regarding systemic immunosuppression.

A similar point applies to the skin weight results. Although both EEBB and DEX significantly reduced the elevated skin weight in the CTL group, this parameter likely reflected inflammatory edema and overall skin thickening rather than epidermal changes alone. Notably, the skin weight in the DEX-treated mice fell slightly below that of the NOR group. This pattern may be consistent with an atrophy-related effect of DEX, a known adverse effect of topical corticosteroids [6], but direct morphometric evidence of skin atrophy was not obtained in the present study. Accordingly, this finding should be interpreted cautiously.

To elucidate the molecular basis of the anti-inflammatory effects of EEBB, we investigated its impact on key inflammatory mediators (NO and PGE_2_) as well as their associated biosynthetic enzymes, iNOS and COX-2, in LPS-stimulated RAW 264.7 macrophages. Prior to these analyses, an MTT assay confirmed that EEBB did not exhibit significant cytotoxicity in RAW 264.7 cells at concentrations up to 400 μg/mL, indicating that the observed anti-inflammatory effects were not attributable to reduced cell viability (Supplementary data S2, Figure S2). Different LPS concentrations were used according to the experimental endpoint: 100 ng/mL for NO and PGE_2_ assays, and 1 μg/mL for Western blot analysis of iNOS, COX-2, and MAPK phosphorylation. This endpoint-specific design was adopted because 100 ng/mL was sufficient to induce measurable production of soluble inflammatory mediators, whereas 1 μg/mL provided more robust and reproducible detection of protein expression and signaling activation.

NO and PGE_2_ serve as direct inflammatory effector molecules responsible for inducing erythema and pain [27]. NO, generated by iNOS, contributes to vasodilation and oxidative stress, whereas PGE_2_, synthesized via COX-2, is a pivotal mediator of increased vascular permeability and pruritus in AD [28]. Our results demonstrated that EEBB dose-dependently and significantly inhibited the LPS-induced production of NO and PGE_2_ (Figure 6), while concurrently downregulating the protein expression of iNOS and COX-2 (Figure 7A). These findings suggest that EEBB suppresses inducible inflammatory mediator production at least in part through regulation of upstream inflammatory signaling, rather than merely reducing end-product mediator levels. Because iNOS and COX-2 are functionally interrelated and can amplify inflammatory responses [29], their coordinated downregulation by EEBB may contribute to the overall anti-inflammatory profile observed in vitro.

The MAPK pathway is a central signaling axis that links extracellular inflammatory stimuli to intracellular transcriptional responses [30]. Given the inhibitory effects of EEBB on iNOS and COX-2, we next examined whether MAPK signaling was involved. Consistent with this hypothesis, our results demonstrated that EEBB significantly suppressed the phosphorylation of p38 and JNK at the highest tested concentration, whereas ERK phosphorylation was not significantly altered (Figure 7B). This differential pattern is mechanistically noteworthy because JNK and p38 are more closely linked to stress- and inflammation-responsive MAPK signaling, whereas ERK is often involved in broader cellular functions, including proliferation, survival, and differentiation [31]. Thus, the selective inhibition of JNK/p38 without a marked effect on ERK suggests that EEBB may preferentially modulate the inflammatory arm of the MAPK network rather than broadly suppressing MAPK signaling as a whole.

JNK and p38 are activated by phosphorylation in response to inflammatory cytokines, growth factors, and environmental stressors, and they are closely associated with transcription factors that regulate iNOS and COX-2 [32,33]. Our findings therefore support the interpretation that suppression of JNK/p38 signaling contributes to the reduced expression of iNOS and COX-2 and to the decreased production of NO and PGE_2_ in LPS-stimulated macrophages. By contrast, the lack of a significant change in ERK phosphorylation suggests that EEBB did not indiscriminately block all MAPK-dependent cellular responses under the present experimental conditions. The relative preservation of ERK signaling may reflect a degree of pathway selectivity, suggesting that EEBB preferentially modulates inflammation-associated MAPK signaling rather than broadly suppressing MAPK activity. However, the biological implications of this apparent selectivity require further investigation.

Interestingly, Yoshida et al. reported that an extract (1 mg/mL) from *Bidens pilosa* L.—a closely related species in the same genus—inhibited COX-2 and PGE_2_ in IL-1β-stimulated fibroblasts, while also suppressing the phosphorylation of p38, JNK, and ERK (albeit to a lesser extent) in the upstream MAPK pathway [34]. This discrepancy between our findings and those of Yoshida et al. may be attributed to variations in harvesting, preparation, and extraction procedures, which result in distinct patterns of bioactive compounds and, consequently, divergent pharmacological actions.

The multi-target efficacy of EEBB is likely attributable to the synergistic effects of its primary bioactive constituents, particularly flavonoids. Quercetin has been extensively documented to attenuate the expression of inflammatory enzymes through the inhibition of signaling pathways [35], while okanin and phenolic components have demonstrated significant activity in modulating cytokine production [14,36,37]. Accordingly, the observed activity of EEBB may reflect the combined actions of multiple phytochemicals rather than the effect of a single dominant constituent.

Despite these promising findings, certain limitations of the present study must be acknowledged. First, although the MC903-induced model reproduces several hallmarks relevant to AD, it remains an experimental AD-like dermatitis model and does not fully capture the entire pathophysiological spectrum of human AD. Second, although immunohistochemical analysis of filaggrin and loricrin provided additional evidence supporting the effects of EEBB on epidermal differentiation- and barrier-associated proteins, direct functional assessments of epidermal barrier integrity, such as TEWL or permeability assays, were not performed. Therefore, the barrier-related effects of EEBB should be interpreted within the scope of the present structural, hydration-related, and protein expression data and should not be taken as direct evidence of functional barrier recovery. Future studies incorporating functional barrier assays will be required to determine whether these changes translate into measurable restoration of epidermal barrier function. Moreover, although pruritus is a major clinical feature of AD, scratching behavior was not quantified in the present study. Therefore, the current findings should not be interpreted as direct evidence of an antipruritic effect of EEBB, and future studies incorporating behavioral itch assessments will be required to determine whether the observed anti-inflammatory and barrier-associated improvements translate into measurable reductions in pruritus-related responses. Additionally, the multi-target nature of EEBB implies that it may exert anti-inflammatory effects through signaling pathways beyond the MAPK axis, which warrants further exploration. Further studies are also needed to identify the major active constituents of EEBB and to clarify how constituent composition relates to pharmacological activity. Future studies employing multi-omics approaches, such as microbiome analysis, are also necessary to fully elucidate the complex mechanisms underlying the therapeutic effects of EEBB in AD-like dermatitis. Specifically, additional investigations into the impact of EEBB on the skin microbiota, functional epidermal barrier integrity, and pruritus-related behavior will be required to provide a more comprehensive understanding of its pharmacodynamic mechanisms.

## 4. Materials and Methods

### 4.1. Medicinal Herb

The herbal material of *B. bipinnata* was collected from the vicinity of the Nakdong River in Yangsan-si, Gyeongsangnam-do, Republic of Korea (35°19′18.2″ N, 129°01′10.0″ E). The plant was authenticated by Prof. Jung-Hoon Kim, and a voucher specimen (No. MH-2024001) has been deposited at the Division of Pharmacology, School of Korean Medicine, Pusan National University.

### 4.2. Preparation of the B. Bipinnata Extract

The dried herbal material (50 g) was weighed, briefly washed, and immersed in 500 mL of 70% ethanol. The mixture was sonicated using a sonicator (Hwashin Tech Co., Ltd., Gwangju-si, Gyeonggi-do, Republic of Korea) for 5 min, followed by continuous orbital shaking at room temperature for 24 h. After the first extraction, the supernatant was decanted and reserved. The residue was then re-extracted with a fresh 500 mL of 70% ethanol under the same conditions. The two extracts were combined and filtered using Whatman No. 20 filter paper (Toyo Roshi Kaisha, Ltd., Tokyo, Japan). The filtrate was concentrated under reduced pressure using a rotary evaporator (IlShinBioBase, Yangju-si, Gyeonggi-do, Republic of Korea) and subsequently lyophilized using a freeze dryer (Labconco, Kansas City, MO, USA).

The extraction process was independently repeated five times, using 50 g of the herbal material per batch. Finally, an average of 1.08 g of lyophilized powder was obtained per extraction, corresponding to a mean yield of 2.17 ± 0.41% (*w*/*w*). The ethanol extract of *B. bipinnata* L. (EEBB) was stored at −20 °C, and samples (Nos. MS2024001–5) have been deposited at the Division of Pharmacology, School of Korean Medicine, Pusan National University (Supplementary data S3, Figures S3 and S4).

### 4.3. Animals

Six-week-old male BALB/c mice were purchased from Hana Biotech (Pyeongtaek, Gyeonggi, Republic of Korea). The animals were housed under controlled conditions (22 ± 2 °C, 50% relative humidity, and a 12 h light/dark cycle) with ad libitum access to standard laboratory chow and water. The animals were acclimated to the laboratory environment for one week prior to the experiments. All animal experimental protocols were approved by the Institutional Animal Care and Use Committee (IACUC) of Pusan National University (Approval No. PNU-2024-0195) and were conducted in accordance with the institutional guidelines.

### 4.4. Induction of AD-like Dermatitis and Experimental Design

Mice were randomly assigned to six groups as follows: the normal group (NOR, n = 6), which received no treatment; the control group (CTL, n = 8), consisting of vehicle-treated mice with MC903-induced AD-like dermatitis; three EEBB treatment groups (n = 8 per group), which received 60, 180, or 600 μg/day of EEBB following MC903 application; and the DEX group (n = 8), a positive control group receiving 150 μg/day of dexamethasone.

**Figure 8:**
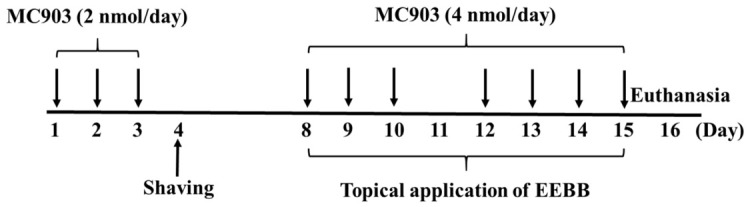
Experimental schedule. EEBB means ethanol extract of *B. bipinnata*.

Except for the NOR group, all animals were sensitized with MC903 (calcipotriol, a vitamin D_3_ analog; MedChemExpress, Monmouth Junction, NJ, USA) dissolved in 100% ethanol for three consecutive days, with 2 nmol/day applied topically to both ears. On day 4, the dorsal hair of each mouse was shaved. After a 3-day stabilization period, the shaved dorsal skin was challenged with MC903 in the morning on 7 days over an 8-day period (3 consecutive days, 1 rest day, and 4 additional consecutive days). During the afternoon of each of these 8 days, EEBB or dexamethasone (DEX; Sigma-Aldrich, St. Louis, MO, USA) was topically applied once daily to the same dorsal skin site. EEBB was prepared as a 50 mg/mL stock solution in 70% ethanol and then diluted with acetone:olive oil (4:1, *v*/*v*) to final concentrations of 1, 3, and 10 mg/mL. DEX was prepared in the same manner. A total volume of 60 μL was applied per mouse per day, corresponding to EEBB doses of 60, 180, and 600 μg/day and a DEX dose of 150 μg/day. The experimental procedure is summarized in Figure 8.

### 4.5. Assessment of Clinical Skin Symptoms and Lesion Scores

On the final day of the experiment (Day 16), mice were anesthetized via intraperitoneal injection of Rompun (Elanco Korea, Seoul, Republic of Korea) and ketamine (Yuhan Corporation, Seoul, Republic of Korea). The dorsal skin of all mice was photographed using a digital camera (IXUS 990 IS; Canon, Tokyo, Japan). Skin lesions were blindly scored based on the severity of symptoms, including scaling, erythema, roughness, and fissuring. A four-point scale was employed (0 = none, 1 = slight, 2 = mild, and 3 = severe), and the total clinical skin score was calculated by summing the individual scores.

### 4.6. Measurement of Erythema Index and Skin Weight

To evaluate changes in skin coloration, erythema index was measured at three separate affected areas on the dorsal skin of each animal using a dermo-spectrophotometer (DSM II; Cortex Technology, Hadsund, Denmark), following the methodology previously described [18,25]. Subsequently, all mice were euthanized, and a circular skin tissue sample (6 mm in diameter) was collected from each mouse using a biopsy punch (HB0926; HEBU Medical, Berlin, Germany). The samples were weighed using a microbalance (Sartorius AG, Göttingen, Germany).

### 4.7. Relative Skin Hydration Readings and Post-Application Moisture Retention Profile

Relative skin hydration readings were measured on Day 16 using a skin hydrometer (Scalar Corp., Tokyo, Japan). For each mouse, measurements were obtained from three non-overlapping dorsal areas. To assess the post-application moisture retention profile, a gauze pad soaked in distilled water was applied to the dorsal skin for 30 s. After excess surface water was gently removed, relative skin hydration readings were recorded at 30 s intervals. A total of four sequential measurements were obtained, and the post-application moisture retention profile was evaluated based on temporal changes in hydration values. All procedures were conducted under controlled environmental conditions (23–24 °C and 40% humidity).

### 4.8. Histopathological Examination, Severity Score Evaluation, and Immune Cell Infiltration Assessment

Excised mouse dorsal skin tissue samples were fixed and embedded in paraffin. Serial sections were cut at a uniform thickness of 4 μm and subjected to hematoxylin and eosin (H&E) staining. Histopathological changes were evaluated under a light microscope at 100× magnification (Carl Zeiss AG, Oberkochen, Germany). To determine the severity score, four non-overlapping microscopic fields per skin tissue sample were assessed. The severity of spongiosis, hyperkeratosis, and basal layer disruption, along with the degree of angiogenesis, were graded on a scale of 0 to 3 (0 = normal; 1 = slight; 2 = moderate; 3 = severe) [18,25].

Quantitative assessment of immune cell infiltration was performed using ZEN imaging software (ZEN 3.0; Carl Zeiss AG, Jena, Germany). For each tissue section, three randomly selected, non-overlapping fields were analyzed at 200× magnification (field of view: 1.28 mm × 0.96 mm) using a standardized counting grid overlay. Within each field, inflammatory cells (including lymphocytes, eosinophils, and neutrophils) were identified by cell type, counted individually, and summed to yield the total inflammatory cell count per field.

### 4.9. Immunohistochemical Analysis of Filaggrin and Loricrin in Skin Tissue

IHC was performed on murine dorsal skin tissue in accordance with the established protocols of our laboratory. Paraffin-embedded sections were deparaffinized, rehydrated, and subjected to heat-induced epitope retrieval (HIER) via complete immersion in sodium citrate buffer (pH 6.0) using a pressure cooker (Cuckoo Electronics Co., Ltd., Yangsan-si, Gyeongsangnam-do, Republic of Korea) for 15 min. Endogenous peroxidase activity was quenched with 0.3% H_2_O_2_, after which non-specific binding was blocked by incubation with normal goat serum from the VECTASTAIN Elite ABC Kits (Vector Laboratories, Inc., Newark, CA, USA) at 37 °C for 1 h. Sections were subsequently incubated overnight at 4 °C with primary antibodies against Filaggrin (catalog #LS-B13715; LifeSpan BioSciences, Inc., Seattle, WA, USA) and Loricrin (catalog #ab85679; Abcam Inc., Waltham, MA, USA), followed by incubation with the appropriate species-matched secondary antibody for 1 h at room temperature. Subsequently, the sections were incubated with the ABC reagent working solution for 1 h. Immunoreactivity was visualized using a diaminobenzidine (DAB) substrate kit (Vector Laboratories, Inc.). Finally, sections were counterstained with hematoxylin, blueing in 0.3% ammonia, dehydrated through graded ethanol, and coverslipped.

### 4.10. Measurement of Cytokine Levels

Cytokine concentrations in mouse dorsal skin samples were quantified using the Mouse Inflammation Cytometric Bead Array (CBA) Kit (BD Biosciences, San Jose, CA, USA). The harvested dorsal skin tissues were minced and homogenized in protein extraction buffer (Thermo Scientific, Mount Prospect, IL, USA). A total of 50 μg of lysate was used to determine the levels of TNF-α, IFN-γ, IL-5, and IL-4. All experimental procedures were performed according to the manufacturer’s instructions.

### 4.11. Cell Culture

RAW 264.7 mouse macrophage cells were purchased from the Korean Cell Line Bank (Seoul, Republic of Korea). The cells were cultured in Dulbecco’s Modified Eagle Medium (DMEM; Gibco, Grand Island, NY, USA) supplemented with 10% heat-inactivated fetal bovine serum (FBS; Gibco, Grand Island, NY, USA) and 1% penicillin–streptomycin (Gibco, Grand Island, NY, USA). Cultures were incubated at 37 °C in a humidified atmosphere containing 5% CO_2_. Subculturing was performed when the cell density reached approximately 80–90% confluence. During subculturing, cells were detached using a cell scraper and gently dislodged by pipetting. Detached cells were collected, resuspended in fresh complete medium, and seeded into new culture dishes at an appropriate density.

### 4.12. Determination of Nitric Oxide Production

NO production in the culture supernatants was determined using the Griess assay. Briefly, cells were seeded in 24-well plates at a density of 1 × 10^5^ cells/well and cultured overnight to allow cell adherence. Following a 4 h pretreatment with EEBB, the cells were stimulated with LPS (100 ng/mL) for 20 h. Upon completion of incubation, the culture supernatants were collected and centrifuged at 1000× *g* for 5 min. Equal volumes of the culture supernatant and Griess reagent (1% sulfanilamide in 5% phosphoric acid and 0.1% N-(1-naphthyl)ethylenediamine dihydrochloride; 1:1, *v*/*v*) were mixed and incubated. After incubation for 10 min at room temperature in the dark, the absorbance was measured at 540 nm using a microplate reader (SpectraMax iD3; Molecular Devices, San Jose, CA, USA). Nitrite concentrations were calculated based on a sodium nitrite (NaNO_2_) standard curve. All measurements were performed in technical triplicate.

### 4.13. Quantification of PGE_2_ Production

PGE_2_ levels in RAW 264.7 cell culture supernatants were quantified using a commercial enzyme-linked immunosorbent assay (ELISA) kit (R&D Systems, Minneapolis, MN, USA). Culture supernatants were collected as described above. The assay was performed according to the manufacturer’s instructions. Absorbance was measured at 450 nm using the microplate reader (SpectraMax iD3; Molecular Devices, San Jose, CA, USA), and PGE_2_ concentrations were calculated based on a standard curve generated from known concentrations provided in the kit. All measurements were performed in technical triplicate.

### 4.14. Protein Extraction and Western Blot Analysis

To evaluate the protein expression of iNOS and COX-2, RAW 264.7 cells were pretreated with EEBB for 8 h, followed by LPS (1 μg/mL) stimulation for 20 h. Additionally, to analyze the activation of the MAPK signaling pathway, cells were pretreated with EEBB for 10 h and then stimulated with LPS (1 μg/mL) for 30 min.

Protein extraction and Western blot analysis were conducted as previously described by our laboratory [14]. Briefly, total protein was extracted using RIPA buffer supplemented with protease and phosphatase inhibitors. Protein concentrations were determined using the Bradford assay. Equal amounts of protein (20 μg per lane) were separated by SDS-PAGE and transferred onto PVDF membranes. Membranes were blocked with 5% skim milk, after which they were incubated overnight at 4 °C with primary antibodies against iNOS, COX-2, p-JNK, JNK, p-p38, p38, p-ERK, ERK, and β-actin. Following incubation with horseradish peroxidase (HRP)-conjugated secondary antibodies at room temperature for 1 h, protein bands were visualized using a luminescent analyzer (Amersham™ Imager 600; GE Healthcare, Little Chalfont, UK). Detailed information on the antibodies used in this study is provided in the Supplementary Data S4 (Table S1).

### 4.15. Statistical Analysis

All data were processed using Microsoft Excel and analyzed using IBM SPSS Statistics 21.0 (IBM Corp., Armonk, NY, USA, 2012). Single-time-point data were analyzed using one-way analysis of variance (ANOVA) followed by Tukey’s or Games-Howell’s post hoc tests, whereas the post-application moisture retention profile was analyzed using two-way ANOVA. Ordinal score data were analyzed using the Kruskal–Wallis test followed by appropriate post hoc multiple comparisons. Graphs were generated using GraphPad Prism version 8.0.2 for Windows (GraphPad Software Inc., La Jolla, CA, USA). Continuous data are expressed as mean ± standard deviation (SD), whereas ordinal score data are presented as median (IQR). A *p*-value of <0.05 was considered statistically significant.

## 5. Conclusions

In this study, topical application of EEBB alleviated MC903-induced AD-like cutaneous symptoms in mice, accompanied by improved skin hydration and post-application moisture retention, as well as partial restoration of filaggrin and loricrin expression in lesional skin. EEBB also attenuated histopathological abnormalities and significantly suppressed the production of pro-inflammatory cytokines (TNF-α and IFN-γ) and Th2 cytokines (IL-4 and IL-5) in skin tissue. In LPS-stimulated RAW 264.7 macrophages, EEBB inhibited NO and PGE_2_ production by downregulating iNOS and COX-2 expression and was associated with reduced phosphorylation of JNK and p38 MAPK, without a significant effect on ERK activation under the present experimental conditions. Collectively, these findings suggest that EEBB may have potential as a candidate for the management of AD-like inflammatory skin conditions, particularly by improving hydration-related parameters and barrier-associated epidermal changes.

## Data Availability

The data presented in this study are available upon request from the corresponding author.

## Supplementary Materials

The following supporting information can be downloaded at: https://www.mdpi.com/article/10.3390/ijms27135717/s1.
