# Supplementary material for: Anti-Inflammatory and Barrier-Related Effects of Bidens bipinnata L. Fruit Ethanol Extract in an MC903-Induced AD-like Dermatitis Mouse Model and LPS-Stimulated RAW 264.7 Cells
Source: Int J Mol Sci. 2026 Jun 24;27(13):5717. doi: 10.3390/ijms27135717 (PMC13361290; doi:10.3390/ijms27135717)
Supplement: Supplementary file 1 [file ijms-27-05717-s001.zip › Supplementary data S2. cytotoxicity assay.pdf]

Supplementary data S2

1. Materials and methods

1.1. Evaluation of Cytotoxic Effects of EEBB

The cytotoxic effects of EEBB on cells were evaluated using the 3-(4,5-dimethylthiazol-2-yl)-2,5-diphenyltetrazolium bromide (MTT) assay. Briefly, RAW 264.7 cells were seeded at a density of 1×10^5 cells/well in 24-well plates, and EEBB was added to each well at concentrations ranging from 0 to 400 μg/mL in complete DMEM. After 4 h of incubation, the culture medium was removed and replaced, and 0.4 mL of MTT solution (5 mg/mL) was added to each well. The plates were then incubated for an additional 3 h. The resulting formazan crystals were dissolved in 0.2 mL of DMSO. Optical density was measured at 540 nm.

2. Result

2.1. EEBB did not exhibit cytotoxicity in RAW 264.7 cells

EEBB did not show cytotoxicity up to dose of 400 μg/mL in RAW 264.7 cells (Figure S2). For this reason, we conduct consecutive experiments using the dose of 0, 25, 50, 100 and 200 μg/mL.

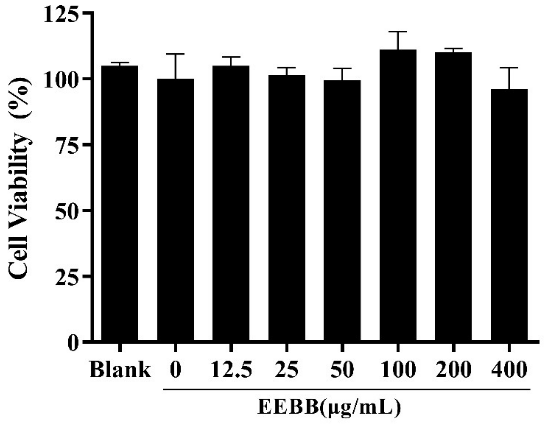

Figure S2. The cytotoxic effects of EEBB on RAW 264.7 cells. Cells were treated with indicated concentrations of EEBB for 4h. All values were represented as mean ± SD of three independent experiments. Blank, untreated group; EEBB, ethanol extract of *Bidens bipinnata* L.
