# Supplementary material for: Anti-Inflammatory and Barrier-Related Effects of Bidens bipinnata L. Fruit Ethanol Extract in an MC903-Induced AD-like Dermatitis Mouse Model and LPS-Stimulated RAW 264.7 Cells
Source: Int J Mol Sci. 2026 Jun 24;27(13):5717. doi: 10.3390/ijms27135717 (PMC13361290; doi:10.3390/ijms27135717)
Supplement: Supplementary file 1 [file ijms-27-05717-s001.zip › Supplementary data S3. HPLC and TP.pdf]

Supplementary data S3

Bidens bipinnata L. (family Asteraceae) is an annual herbaceous plant widely distributed across tropical, subtropical, and temperate regions. The whole plant of *B. bipinnata* has been used in China, Japan, and Korea to treat patients with various inflammatory and infectious conditions, and is traditionally applied topically to treat skin infections, boils, and venomous insect bites. In the theory of traditional medicine, *B. bipinnata* is characterized by a cooling nature and is classified as a remedy to clear away superficial heat and toxic materials, eliminate wind-dampness, and resolve blood stasis. The herbal material used in this study was collected from the vicinity of the Nakdong River in Yangsan-si, Gyeongsangnam-do, Republic of Korea (35°19'18.2"N, 129°01'10.0"E).

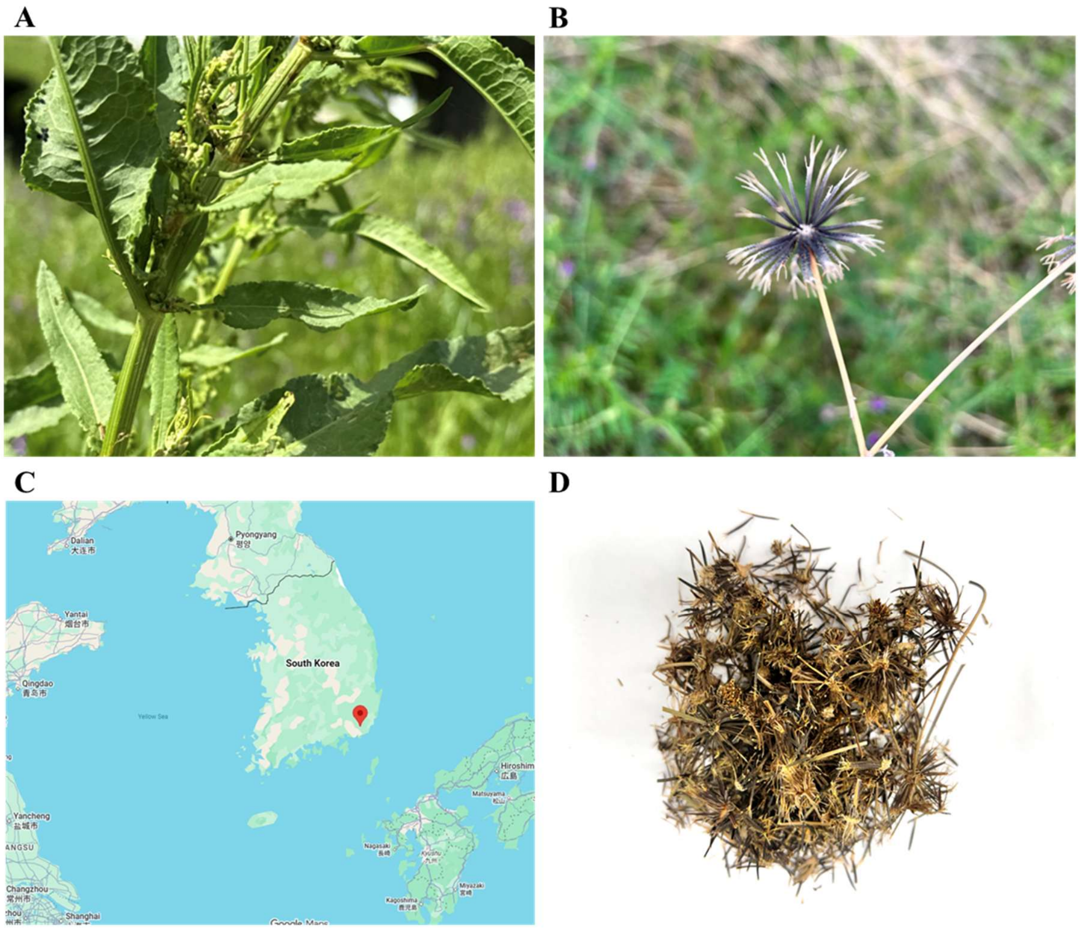

Figure S3. *Bidens bipinnata L.* The leaves (A), the fruits (B), collection site (C) and dried fruits of *B. bipinnata* (D).

Component verification through HPLC

1. Materials and methods

1.1. Chemicals and reagents

HPLC-grade methanol, acetonitrile, and water were purchased from J.T. Baker Inc. (Phillipsburg, USA). Trifluoroacetic acid (TFA), Folin–Ciocalteu's phenol reagent, Sodium carbonate, Sodium nitrite, Aluminum chloride hexahydrate and sodium hydroxide were purchased from Sigma-Aldrich (St

Louis, MO, USA). Luteolin 7-glucuronide was purchased from Chengdu Biopurify Phytochemicals Ltd. (Chengdu, Sichuan, China). Gallic acid was purchased from ChemFaces Ltd. (Wuhan, Hubei, China). Rutin was purchased from Agilent Technologies Ltd. (Santa Clara, California, USA).

1.2. Determination of Total Polyphenol and Flavonoid Contents

The total phenolic content (TPC) of EEBB samples was determined using the Folin–Ciocalteu assay, adapted from the method described by Haroon Rasheed[1]. Briefly, 20 μL of appropriately diluted samples or standard solutions were mixed with 100 μL of a freshly prepared 10-fold diluted Folin–Ciocalteu reagent. After a 5 min reaction, the mixture was neutralized by adding 80 μL of saturated sodium carbonate solution (75 g/L). Following a 90 min incubation in the dark at room temperature, the absorbance was measured at 725 nm using a microplate reader (SpectraMax iD3; Molecular Devices, USA). All measurements were performed in duplicate to ensure reproducibility. Gallic acid served as the standard, and the TPC was expressed as milligrams of gallic acid equivalent per gram of dry extract weight (mg GAE/g extract).

The total flavonoid content (TFC) was determined using the aluminum chloride colorimetric method adapted from Haroon Rasheed [1]. Briefly, 100 μL of appropriately diluted EEBB or standard solutions were mixed with 15 μL of 5% (w/v) $NaNO_2$. After a 5-min incubation, 15 μL of 10% (w/v) $AlCl_3·6H_2O$ was added, and the mixture was allowed to react for 6 min. Subsequently, 100 μL of 1 M NaOH and 300 μL of ddH_2O were sequentialy introduced. The reaction mixture was then incubated in the dark at room temperature for 45 min, after which the absorbance was measured at 510 nm using a microplate reader. All samples were analyzed in duplicate. Rutin served as the standard, and the TFC was expressed as milligrams of rutin equivalent per gram of dry extract weight (mg RE/g extract). TPC: total phenolic content expressed as mg GAE/g extract; TFC: total flavonoid content expressed as mg RE/g extract.

1.3. Preparation of standard solution and sample solution

Luteolin 7-glucuronide was accurately weighed and dissolved in methanol at the concentrations of 1000 μg/mL to make a standard solution. Sample (EEBB) was diluted with the HPLC-grade methanol and then filtered through a 0.2 μm syringe filter (BioFACT™, Daejeon, Republic of Korea) prior to HPLC injection.

1.4. Chromatography and mass spectrometry

Qualitative analysis was performed using an Agilent 1200 series HPLC system (Agilent Technologies, Santa Clara, CA, USA) and data were acquired using ChemStation software (Agilent Technologies). A Capcell Pak Mg II C18 column (4.6 mm × 250 mm, 5 μm; Shiseido, Tokyo, Japan) was used for the separation of analytes in a column oven at 35 °C. The flow rate was set at 1 mL/min and the injection volume was set at 10 μL. A gradient elution system was applied with the mobile phase consisting of water containing 0.1% TFA (A) and acetonitrile (B): 5% (B) over 0–8 min, 12% (B) over 8–18 min, 30% (B) over 18–25 min, 70% (B) over 25–30 min, 75% (B) over 30–33 min, and 5% (B) over 33–36 min. The detection wavelength was set at UV 350 nm.

2. Results

2.1. Total Polyphenol and Total Flavonoid Contents of the Sample

The total phenolic and flavonoid contents of the EEBB extract were determined using gallic acid and rutin as standards, respectively. The total phenolic content was 152.82 mg GAE/g extract, whereas the total flavonoid content was 134.1 mg RE/g extract.

2.2. Identification and Quantification of luteolin 7-glucuronide in the sample

The peak of luteolin 7-glucuronide detected in the sample (tR: 15.403 min) was identified by comparison of its retention time and UV spectrum with those of the authentic standard. Quantitative

analysis was then performed using an HPLC calibration curve for luteolin 7-glucuronide ($y = 31.884x + 73.279$, $R^2 = 0.9991$), and the content in EEBB was determined to be approximately 16.4 ppm based on the observed peak area (Figure S4).

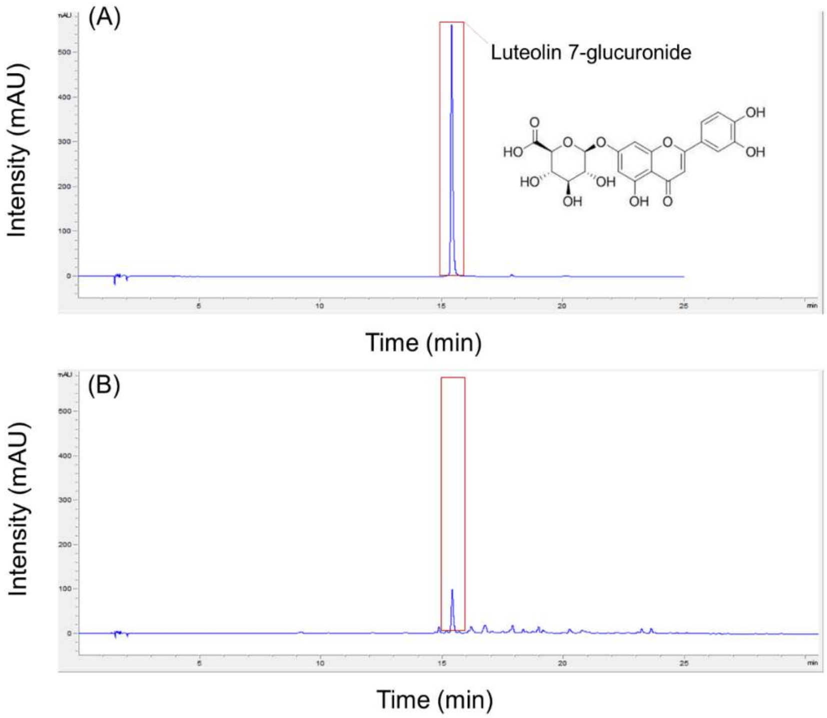

Figure S4. Chromatograms of the luteolin 7-glucuronide standard (A) and the EEBB sample solution (B) detected at 350 nm.
