# Supplementary material for: Anti-Inflammatory and Barrier-Related Effects of Bidens bipinnata L. Fruit Ethanol Extract in an MC903-Induced AD-like Dermatitis Mouse Model and LPS-Stimulated RAW 264.7 Cells
Source: Int J Mol Sci. 2026 Jun 24;27(13):5717. doi: 10.3390/ijms27135717 (PMC13361290; doi:10.3390/ijms27135717)
Supplement: Supplementary file 1 [file ijms-27-05717-s001.zip › Supplementary data S4. The Antibody sets for Western Blot.pdf]

**Supplementary data S4**

Table S1. Antibody information used for Western Blot in this study

| Name | primary antibody information | Dilution |
|---|---|---|
| iNOS | #13120, Cell Signaling Technology, Danvers, MA, USA | 1:1000 |
| COX-2 | #sc-376861, Santa Cruz Biotechnology, Dallas, TX, USA | 1:1000 |
| P-ERK | #4370, Cell Signaling Technology, Danvers, MA, USA | 1:2000 |
| ERK2 | #4695, Cell Signaling Technology, Danvers, MA, USA | 1:1000 |
| P-p38 | #4631, Cell Signaling Technology, Danvers, MA, USA | 1:1000 |
| p38 | #9212, Cell Signaling Technology, Danvers, MA, USA | 1:1000 |
| p-JNK | #4671, Cell Signaling Technology, Danvers, MA, USA | 1:1000 |
| JNK | #9258, Cell Signaling Technology, Danvers, MA, USA | 1:1000 |
| β-actin | #sc-47778, Santa Cruz Biotechnology, Dallas, TX, USA | 1:1000 |
| | Secondary antibody information | |
| | Goat anti-mouse IgG F(ab')2, polyclonal antibody (HRP conjugate), Enzo Life Sciences, Farmingdale, NY, USA | 1:2000 |
| | Goat anti-rabbit IgG F(ab')2, polyclonal antibody (HRP conjugate), Enzo Life Sciences, Farmingdale, NY, USA | 1:2000 |
