# Supplementary material for: Anti-Inflammatory and Barrier-Related Effects of Bidens bipinnata L. Fruit Ethanol Extract in an MC903-Induced AD-like Dermatitis Mouse Model and LPS-Stimulated RAW 264.7 Cells
Source: Int J Mol Sci. 2026 Jun 24;27(13):5717. doi: 10.3390/ijms27135717 (PMC13361290; doi:10.3390/ijms27135717)
Supplement: Supplementary file 1 [file ijms-27-05717-s001.zip › Supplementary data S1. Spleen body weight ratio.pdf]

Supplementary data S1

1. Materials and methods

1.1. Measurement of body and spleen weights

Initial and final body weights were recorded on days 1 and 16, respectively. Upon euthanasia on day 16, spleen weights were obtained using a microbalance (Sartorius AG, Göttingen, Germany). Weight gain or loss was calculated as a percentage of the initial body weight. To evaluate the systemic immune response, the spleen index was determined by calculating the ratio of spleen weight to total body weight.

2. Result

2.1. EEBB did not affect spleen enlargement

To evaluate the impact of EEBB on splenomegaly, the spleen index (spleen-to-body weight ratio) was calculated. The spleen indices of the groups receiving EEBB were comparable to those of the CTL group. However, treatment with DEX resulted in a significant reduction in the spleen index (Figure S1).

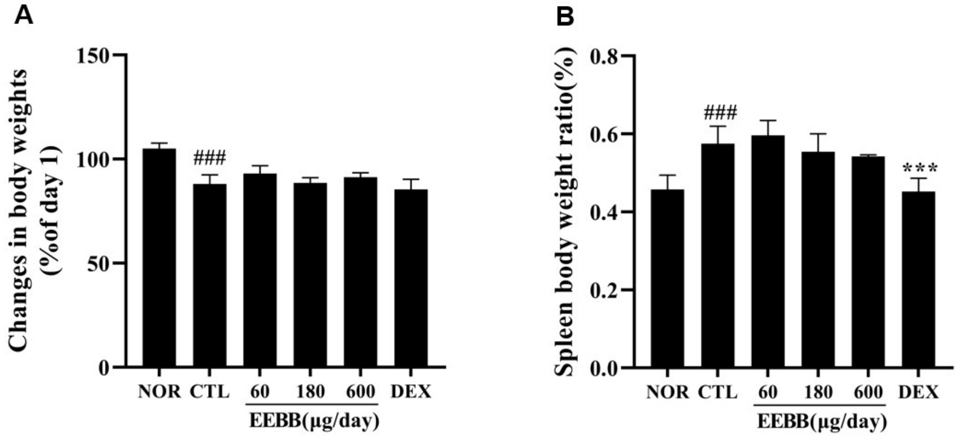

Figure S1. Effects of EEBB on spleen/body weight ratio in AD mice. Body and spleen weight were measured on day 16 and the spleen/body weight ratio was calculated. (A), changes in body weights; (B), spleen/body weight ratio. EEBB, ethanol extract of *Bidens bipinnata* L.; DEX, dexamethasone. ###*P* < 0.001 vs. NOR; ****P* < 0.001 vs. CTL.
